# Integrative host–microbiome modeling uncovers the implication of oral–gut translocation in advanced cirrhosis

**DOI:** 10.1002/imt2.70131

**Published:** 2026-05-07

**Authors:** Yi Jin, Frederick Clasen, Fernando Garcia‐Guevara, Sania Arif, Robert Schierwagen, Gholamreza Bidkhori, Michael Praktiknjo, Maximilian J. Brol, Frank E. Uschner, Florence A. Castelli, Nicolas Pons, Benoit Quinquis, Nathalie Galleron, Kevin Da Silva, Christophe Junot, Debbie L. Shawcross, David L. Moyes, Rajiv Jalan, S. Dusko Ehrlich, Vishal C. Patel, Jonel Trebicka, Saeed Shoaie

**Affiliations:** ^1^ Centre for Host‐Microbiome Interactions, Faculty of Dentistry, Oral & Craniofacial Sciences, King's College London London UK; ^2^ Department of Internal Medicine B University of Münster Münster Germany; ^3^ CEA, INRAE, Département Médicaments et Technologies pour la Santé (MTS), MetaboHUB‐IDF Université Paris‐Saclay Gif‐sur‐Yvette France; ^4^ Université Paris‐Saclay, INRAE, MGP Jouy‐en‐Josas France; ^5^ Roger Williams Institute of Liver Studies, School of Immunology and Microbial Sciences, Faculty of Life Sciences and Medicine, King's College London London UK; ^6^ Liver Failure Group, UCL Institute for Liver and Digestive Health London UK; ^7^ European Foundation for the Study of Chronic Liver Failure, EF CLIF Barcelona Spain; ^8^ Department of Clinical and Movement Neurosciences UCL Queen Square Institute of Neurology, University College London London UK; ^9^ Department of Medical Gastroenterology and Hepatology University of Southern Denmark Odense Denmark; ^10^ Quantitative Systems Biology, Faculty of Medicine, Biruni University Istanbul Turkey

## Abstract

Liver cirrhosis is associated with profound disruption of host–microbiome metabolic interactions. Using paired oral and fecal metagenomics combined with genome‐scale metabolic modeling, we investigated how microbial translocation along the oral–gut axis influences microbial metabolism at different cirrhosis severities. Reactobiome‐based functional profiling revealed progressive metabolic convergence between oral and gut microbiomes, quantified by a decrease in oral–gut metabolic distance. Translocation‐associated microbial species enriched in patients with cirrhosis were predicted to have elevated capacities for ammonia and acetate production. Microbial‐community and host metabolic modeling further suggested that these microbial metabolic shifts may influence host energy metabolism and redox balance across the liver, brain, and skeletal muscle. Together, these findings suggest a potential acetate‐ammonia metabolic axis linking oral–gut microbial translocation with systemic metabolic stress in advanced cirrhosis.

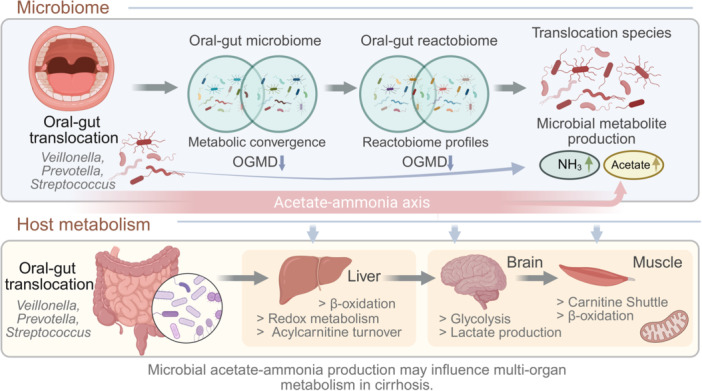

To the editor,

Liver cirrhosis is a systemic metabolic disorder in which host physiology, microbial ecosystems, and organ‐level metabolism become profoundly dysregulated [1, 2]. In addition to well‐recognized gut microbial dysbiosis, recent metagenomic studies have reported the presence of oral‐associated bacteria, including *Streptococcus*, *Veillonella,* and *Prevotella*, in the gut microbiome of patients with cirrhosis [3, 4, 5]. This oralisation of the gut microbiome suggests microbial transmission along the oral–gut axis during cirrhosis progression [6]. However, despite the recognition of oral–gut microbial translocation, most studies have focused primarily on taxonomic composition, leaving the functional metabolic consequences of these cross‐site microbial invasions largely unexplored [7, 8].

The liver is continuously exposed to gut‐derived microbial metabolites and products through the portal circulation, making hepatic physiology highly sensitive to microbial metabolic activity [9, 10]. In cirrhosis, microbes in the gut show reduced short‐chain fatty acid production and increased amino‐acid and nitrogen metabolism, processes closely linked to hyperammonaemia and hepatic decompensation [11]. However, whether translocated species contribute to this metabolic perturbation has not been described. Addressing this possibility requires integrative approaches that link microbial community structure with metabolic activity across the oral–gut–liver axis.

## REACTOTYPING REVEALS SEVERITY‐ASSOCIATED METABOLIC ORGANIZATION AND ORAL–GUT FUNCTIONAL CONVERGENCE

**Figure 1:**
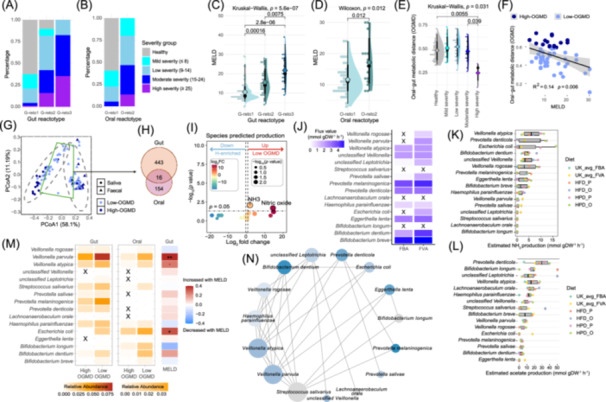
Reactotype‐associated metabolic convergence between oral and gut microbiomes in liver cirrhosis. Distribution of cirrhosis severity (MELD score categories) across gut (A) and oral (B) reactotypes. (C) MELD scores across gut reactotypes (Kruskal–Wallis test with pairwise Wilcoxon tests). (D) MELD scores between oral reactotypes (Wilcoxon rank‐sum test). (E) Oral–gut metabolic distance (OGMD) across healthy controls and cirrhosis severity groups (Kruskal–Wallis test with pairwise Wilcoxon rank‐sum tests). (F) Correlation between OGMD and MELD score. Patients were stratified into high‐ and low‐OGMD groups based on the 75th percentile. (G) Principal coordinates analysis (PCoA) of oral and gut reactobiome, colored by OGMD group (threshold: 75th percentile) and shaped by sample type. (H) Identification of 16 tMSPs, representing oral‐gut overlapping species with higher mean abundance in low‐OGMD patients in the gut (459 species) and oral (170 species) microbiomes. (I) Volcano plot comparing predicted metabolite production between gut commensals and the 16 tMSPs (Wilcoxon rank‐sum test). NH_3_ and nitric oxide production were predicted to be higher in tMSPs. (J) Predicted NH_3_ production fluxes of the 16 tMSPs inferred from genome‐scale metabolic models using flux balance analysis (FBA) and flux variability analysis (FVA). Predicted ammonia (K) and acetate (L) production fluxes of GSMMs under five dietary regimes (UK average diet, high‐fiber diet, high‐protein diet, omnivorous, plant‐based). (M) Relative abundance of the 16 tMSPs in oral and gut microbiomes and their correlations with MELD score (Spearman correlation, **0.001 ≤ FDR < 0.01; *0.01 ≤ FDR < 0.05; • 0.05 ≤ FDR < 0.1). (N) Co‐abundance network of the 16 tMSPs in the gut microbiome based on significant Spearman correlations (FDR < 0.05). Node size corresponds to the degree score within the network, and node color reflects the predicted NH_3_ production of each species. MELD, Model for End‐Stage Liver Disease; OGMD, oral–gut metabolic distance; tMSPs, translocation‐associated metagenomic species pangenomes.

Reactobiome‐based functional profiling, which captures the collective metabolic reaction repertoire encoded by microbiome genes [12], revealed that microbial metabolic organization stratifies cirrhosis severity across both gut and oral ecosystems. Unsupervised clustering of reaction abundance matrices identified three gut reactotypes (G‐reto1‐3) and two oral reactotypes (O‐reto1‐2), which followed a clear severity gradient (Figure 1A,B, Figure S1, Table S1). G‐reto1 was dominated by healthy individuals (61.5%) and patients with mild disease (35.6%), whereas G‐reto3 was enriched in moderate (61.5%) and high‐severity (23.1%) cases. Consistently, Model for End‐Stage Liver Disease (MELD) scores increased progressively across gut reactotypes (Kruskal–Wallis, *p* = 5.6 × 10^−7^; Wilcoxon rank‐sum test, *p* = 2.8 × 10^−6^) (Figure 1C). Similarly, O‐reto2 showed significantly higher MELD scores than O‐reto1 (Wilcoxon rank‐sum test, *p* = 0.012) (Figure 1D). These associations were independent of age and BMI across reactotypes (Figure S2).

We then quantified oral–gut functional divergence using the Oral–Gut Metabolic Distance (OGMD), defined as the Bray–Curtis dissimilarity between oral and gut reactobiome profiles. OGMD decreased stepwise across cirrhosis severity groups (Kruskal–Wallis, *p* = 0.031) and showed a significant negative association with MELD score (*R* ^2^ = 0.14, *p* = 0.006), indicating progressive metabolic convergence between oral and gut microbiomes during cirrhosis progression (Figure 1E,F).

Gut reactotypes associated with more severe disease were enriched in peptidoglycan biosynthesis and nucleotide metabolism, whereas milder states were enriched in aminoacyl‐tRNA biosynthesis and carbon fixation pathways (Figure S3). Stratifying individuals by OGMD further identified pathways associated with oral‐gut functional overlap. Low‐OGMD patients were enriched in teichoic acid biosynthesis, peptidoglycan metabolism, and terpenoid backbone biosynthesis, while pathways involved in branched‐chain amino‐acid degradation and butanoate metabolism were depleted (Figure S4). These patterns were independent of clinical metadata, including disease etiology (Figure S5).

## TRANSLOCATING ORAL‐ASSOCIATED SPECIES ENHANCED AMMONIA‐PRODUCING METABOLIC POTENTIAL

Principal coordinates analysis (PCoA) of paired oral and gut reactobiome profiles visualized the OGMD‐based stratification, with low‐OGMD patients located near where PCoA axis 1 approached zero (Figure 1G). Given reported oral–gut microbial translocation in advanced cirrhosis and the role of taxonomic composition in shaping microbiome metabolic function, translocation‐associated species linked to the high metabolic convergence state were examined. Using OGMD as a continuous metric, we identified species showing a trend towards higher abundance in low‐OGMD patients separately in gut and oral microbiomes and defined 16 translocation‐associated metagenomic species pangenomes (tMSPs) as the overlapping subset detected in both sites (Figure 1H). Consistently, the abundance of these tMSPs showed a significant negative association with OGMD (*p* < 0.05), in contrast to the remaining oral–gut overlapping species (*n* = 19) and other MSPs, which displayed a positive association (Figure S6). To investigate their metabolic capacity, we then used genome‐scale metabolic models (GSMMs) in silico flux simulations [13].

Flux balance analysis (FBA) predicted that the 16 tMSPs have significantly higher capacities for ammonia (NH_3_) and nitric oxide (NO) production based on a UK‐average diet compared to a set of 26 gut commensal species that were prevalent and enriched in healthy control samples (Wilcoxon rank‐sum test, *p* < 0.05, Figure 1I). Among the 16 tMSPs, 10 were predicted to produce NH_3_, compared with 7 of the 26 gut commensals (Figure 1J, Figure S7, Table S2). Flux variability analysis, which evaluates the range of feasible fluxes under the same optimal objective as opposed to a single solution in FBA, further confirmed ammonia production capacity in 13 tMSPs across feasible flux ranges under different nutrient scenarios (Figure 1K). In addition, metabolic simulations revealed that several tMSPs also produced acetate, with five species, including *Prevotella denticola* and *Veillonella atypica*, predicted to co‐produce both ammonia and acetate (Figure 1L), suggesting a combined nitrogen and carbon metabolic burden associated with oral‐gut microbial translocation.

Many tMSPs accumulated preferentially in the gut microbiomes of low‐OGMD patients and were positively associated with cirrhosis severity (Figure 1M). Among the tMSPs with sufficient abundance for reliable quantification, the abundance of *Veillonella parvula*, *Veillonella atypica*, and *Escherichia coli* was positively correlated with MELD score (Spearman, FDR < 0.1). Co‐abundance network of the 16 tMSPs revealed a highly interconnected community where *Prevotella* and *Veillonella* species formed central hubs, with *V. atypica* and *V. parvula* displaying the highest network degree and strong predicted NH_3_ production capacity (Figure 1N, Table S3). Most tMSPs, particularly *Veillonella*, were more prevalent in oral than gut microbiomes, consistent with their oral origin and supporting their role in oral–gut microbial translocation (Figure S7G). Finally, reaction‐level GSMM analysis indicated that ammonia production in tMSPs is primarily derived from amino‐acid metabolism pathways, including L‐asparagine, l‐threonine, l‐cysteine, l‐serine, l‐arginine and l‐glutamine metabolism (Figure S8). Many of these pathways, including arginine, asparagine, and threonine degradation, are known acid‐stress adaptation mechanisms in oral bacteria [14]. Such metabolic flexibility may facilitate niche adaptation and survival of oral microbes during gastrointestinal transit while increasing nitrogen release into the intestinal environment.

## COMMUNITY METABOLIC MODELING REVEALS ECOSYSTEM‐LEVEL AMPLIFICATION OF AMMONIA PRODUCTION

**Figure 2:**
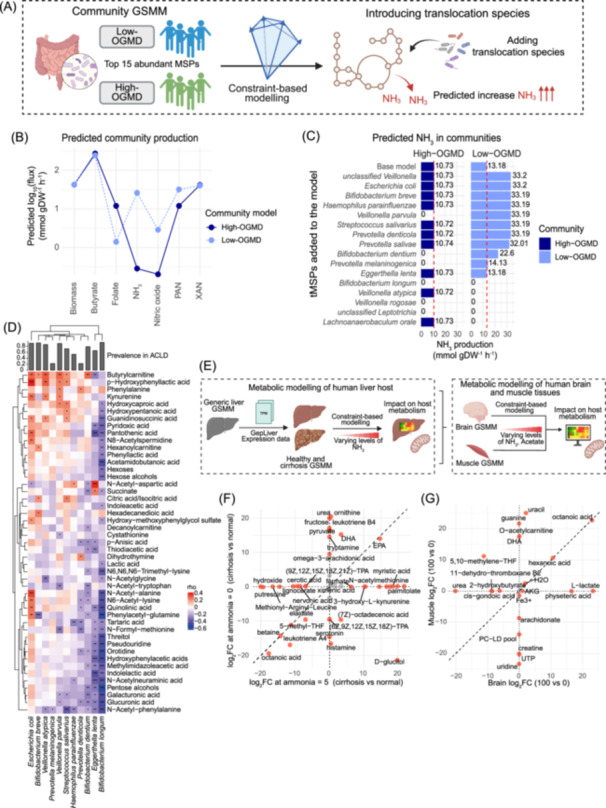
Community and host metabolic modeling linking oral–gut microbial translocation to systemic metabolism. (A) Workflow of community genome‐scale metabolic modeling (GSMM). The top 15 most abundant species from high‐ and low‐OGMD patients were used to reconstruct community models to evaluate the impact of translocation species on ammonia production. (B) Predicted community metabolite production in high‐ and low‐OGMD communities. (C) Predicted NH_3_ production following the introduction of individual translocation species into high‐ and low‐OGMD community models. (D) Associations between tMSP abundance and portal‐vein metabolites in the TIPS cohort. Heatmap shows significant Spearman correlations (FDR < 0.05), and the bar plot indicates the prevalence of each tMSP in the TIPS cohort. (E) Workflow of condition‐specific GSMMs for host tissues. Transcriptomic data from the GepLiver database were integrated into a human liver GSMM to construct condition‐specific liver models, and simulations were performed under increasing ammonia exposure. Brain and muscle GSMMs were simulated under increasing ammonia and acetate exposure. (F) Differential hepatic metabolite production predicted by liver GSMMs comparing cirrhosis and healthy models under high (5 mmol·gDW^−1^·h^−1^; *x*‐axis) and low (*y*‐axis) ammonia uptake. Metabolites with |log_2_FC| ≥ 3.5 are labeled. (G) Differential metabolite production predicted in brain and muscle GSMMs under high versus zero ammonia and acetate exposure (100 vs. 0 mmol·gDW^−1^·h^−1^). Metabolites with |log_2_FC| ≥ 1 are labeled. ACLD, advanced chronic liver disease; OGMD, oral–gut metabolic distance; TPM, transcripts per million.

To determine how translocation‐associated species reshape microbiome metabolic activity, patient‐representative community GSMMs based on species relative abundances were constructed. For each OGMD group, the top 15 most abundant species were used to build average community models for low‐ and high‐OGMD gut microbiomes (Figure 2A). The low‐OGMD community contained five tMSPs among its dominant members, whereas the high‐OGMD community included only one, consistent with the higher prevalence of translocation species in patients with strong oral–gut metabolic convergence. Under identical simulation constraints, both communities achieved comparable biomass production (Figure 2B, Table S4). However, the low‐OGMD community produced higher levels of ammonia and nitric oxide, while showing reduced folate production, indicating a shift toward nitrogen‐centered metabolic activity.

To evaluate the contribution of individual tMSPs to community metabolism, we reconstructed baseline gut community models composed of the top 15 abundant species, excluding the 16 tMSPs, and systematically introduced each tMSP. This perturbation analysis showed that 10 of the 16 tMSPs increased ammonia production when introduced into the low‐OGMD community, whereas the same perturbations produced minimal effects in the high‐OGMD community (Figure 2C, Table S4). This suggested that gut microbiomes from patients with strong oral–gut metabolic convergence exhibit greater modeled sensitivity to species‐level perturbations, reflecting an increased susceptibility of predicted community metabolism to translocating taxa, and potentially consistent with reduced ecological stability. Together, these findings provide a model‐based explanation for the observed stronger functional effects of microbial translocation in advanced cirrhosis with pre‐existing gut dysbiosis.

Taken together, our community modeling suggests that gut microbiomes exhibiting stronger oral–gut metabolic convergence may be more susceptible to metabolic perturbation by invading taxa and that resident gut microbes metabolically interact with oral‐derived species. The co‐production of acetate with ammonia is clinically relevant because previous studies have shown that during alcohol consumption, acetate reprograms gut microbiome, but at the same time, that acetate may also play a role in hyperammonemia and ultimately increase systemic metabolic burden [15, 16].

### Host metabolic modeling suggests potential multi‐organ consequences of microbial ammonia burden

To evaluate the potential clinical relevance of these predictions, we analyzed an independent cirrhosis cohort with stool metagenomics and portal‐vein metabolomics data. Among the 16 tMSPs identified in the discovery dataset, 11 were detected in the independent cohort (Figure 2D, Table S5), with several showing high prevalence, including *V. parvula* (0.89), *V. atypica* (0.86). Correlation analysis between species abundance and portal‐vein metabolites revealed significant associations between tMSPs and metabolic signatures indicative of altered nitrogen and mitochondrial metabolism. Notably, *V. atypica* was positively correlated with guanidinosuccinic acid, a marker of ammonia overload [16], whereas multiple acetate‐producing species were associated with butyrylcarnitine, linked to impaired mitochondrial β‐oxidation (Spearman, FDR < 0.05) [17]. These results provide independent support for the predicted metabolic activity of translocating species and suggest that microbial nitrogen metabolism may contribute to systemic metabolic alterations in cirrhosis.

Given that the liver is the primary organ responsible for ammonia detoxification through ureagenesis, we examined how increased microbial ammonia production might influence host metabolism [10]. Using transcriptome‐constrained liver GSMMs representing healthy and cirrhotic hepatic tissue, we simulated increasing extracellular ammonia uptake (Figure 2E). Comparison of flux distributions revealed substantial metabolic reprogramming in cirrhotic hepatocytes, with the largest changes occurring in lipid and energy metabolism (Figure S9A,B). Flux through fatty‐acid activation, mitochondrial and peroxisomal β‐oxidation, oxidative phosphorylation, carnitine‐dependent transport, and reactive oxygen species detoxification was markedly increased (Figure 2F, Table S6), indicating enhanced energetic and redox demands associated with ammonia detoxification. These predictions suggested portal‐vein metabolomic signals indicative of intensified fatty‐acid oxidation and acylcarnitine turnover [17].

Excess ammonia not detoxified by the liver enters systemic circulation and could be metabolized by peripheral tissues such as skeletal muscle and brain [10, 15]. In addition, circulating acetate in cirrhosis may originate from both hepatic ethanol metabolism and microbial fermentation in the gut [18]. The potential production of acetate and ammonia by translocating microbial species, therefore, introduces an additional metabolic load that may escape hepatic clearance. We therefore evaluated the systemic consequences of ammonia and acetate exposure using brain and skeletal muscle GSMMs (Figure 2E). Under elevated extracellular ammonia and acetate, the brain model predicted increased lactate production, whereas the muscle model showed elevated 2‐hydroxybutyrate and O‐acetylcarnitine production (Figure 2G, Table S6). In both tissues, flux through the carnitine shuttle and mitochondrial β‐oxidation increased, indicating intensified energetic demand and redox stress under combined ammonia‐acetate exposure [19, 20]. These responses parallel the enhanced fatty‐acid oxidation and mitochondrial activity predicted in the cirrhosis liver model, suggesting that microbial acetate and ammonia production may represent a potential driver of systemic alterations in host energy metabolism in cirrhosis [17].

### Application and clinical relevance

We present a functional framework for investigating microbial transmission along the oral–gut axis in advanced cirrhosis. Integrating reactobiome‐based metabolic profiling with GSMM suggests that oral and gut microbiomes become progressively metabolically aligned with increasing disease severity, and that translocation‐associated microbial species may contribute to nitrogen‐centered metabolic dysregulation. Hyperammonemia is one of the major driving factors for the development of hepatic encephalopathy and acts as a biomarker for mortality in people with cirrhosis [10]. Previously, excess circulating ammonia was thought to result mainly from increased gut production and impaired urea‐cycle function [10, 18]. Our work here suggests that the microbiome could be another source of ammonia production, linked with acetate production that also has clinical consequences. This forms the rationale for gut microbiome treatments for hyperammonemia using poorly absorbed antibiotics such as rifaximin and by targeting specific taxa with technologies such as bacteriophage‐based approaches. This will be essential for experimentally validating and translating the modeling predictions presented here. Future studies combining longitudinal sampling, targeted metabolomics, experimental microbiome models, and therapeutic modulation will allow confirmation of the causal contribution of oral–gut translocation to hyperammonemia in cirrhosis.

## AUTHOR CONTRIBUTIONS

**Yi Jin**: Conceptualization; investigation; writing—original draft; methodology; visualization; formal analysis. **Frederick Clasen**: Investigation; writing—review and editing; formal analysis; methodology. **Fernando Garcia‐Guevara**: Methodology; writing—review and editing; investigation; formal analysis. **Sania Arif**: Formal analysis; writing—review and editing. **Robert Schierwagen**: Formal analysis; writing—review and editing. **Gholamreza Bidkhori**: Writing—review and editing; methodology. **Michael Praktiknjo**: Formal analysis; writing—review and editing. **Maximilian J. Brol**: Formal analysis; writing—review and editing. **Frank E. Uschner**: Formal analysis; writing—review and editing. **Florence A. Castelli**: Formal analysis; writing—review and editing. **Nicolas Pons**: Formal analysis; writing—review and editing. **Benoit Quinquis**: Formal analysis; writing—review and editing. **Nathalie Galleron**: Writing—review and editing; formal analysis. **Kevin Da Silva**: Writing—review and editing; formal analysis. **Christophe Junot**: Writing—review and editing; formal analysis. **Debbie L. Shawcross**: Writing—review and editing. **David L. Moyes**: Writing—review and editing. **Rajiv Jalan**: Writing—review and editing. **S. Dusko Ehrlich**: Writing—review and editing. **Vishal C. Patel**: Writing—review and editing. **Jonel Trebicka**: Writing—review and editing. **Saeed Shoaie**: Conceptualization; investigation; writing—review and editing; supervision. All authors have read the final manuscript and approved it for publication.

## CONFLICT OF INTEREST STATEMENT

Frederick Clasen and Saeed Shoaie are co‐founders and shareholders of Gigabiome Ltd. Saeed Shoaie is co‐founder and shareholder of Trustlife Therapeutics. Debbie L. Shawcross declares consultancy roles with Norgine Pharmaceuticals Ltd., Alfa Sigma, EnteroBiotix, MRM Health, GENFIT, Satellite Biosciences, and Apollo Therapeutics Ltd. Vishal C. Patel declares consultancy roles with Resolution Therapeutics, Emles Bioventures, AlfaSigma S.p.A., AstraZeneca, Norgine Pharmaceuticals Ltd, and Menarini Diagnostics Ltd. The remaining authors declare no conflicts of interest.

## ETHICS STATEMENT

All patients provided written informed consent, and the TIPS study was approved by the local ethics committee (University Hospital Bonn, approval no. 203/13).

## Supporting information


**Figure S1.** Determination of gut and oral reactotypes.
**Figure S2.** Association of gut and oral reactotypes with clinical and demographic characteristics.
**Figure S3.** Unique reactions featured in each gut and oral reactotype.
**Figure S4.** Significant reactions distinguishing low‐ and high‐OGMD patients.
**Figure S5.** Clinical metadata and aetiology differences between high‐ and low‐OGMD patients.
**Figure S6.** Linear regression analysis of tMSPs, remaining overlapping MSPs and other MSPs in relation to OGMD.
**Figure S7.** Flux balance and variability analysis of translocation species and healthy‐gut commensals.
**Figure S8.** Metabolic reactions involved in NH_3_ production of tMSPs.
**Figure S9.** Subsystem‐level metabolic differences between cirrhotic and healthy liver GSMMs under increasing NH_3_ concentrations.

**Table S1.** Reaction abundance in the oral and gut reactotypes.
**Table S2.** Bacterial species single modelling results.
**Table S3.** Co‐abundance network results.
**Table S4.** Bacterial community modelling results.
**Table S5.** Species prevalence in the validation cohort.
**Table S6.** Human tissue modelling results.
**Table S7.** Relative abundance of oral and gut species.

## Data Availability

The shotgun metagenomic raw data used in this study are publicly available from the European Nucleotide Archive (ENA) under the project accessions PRJEB52891 (GLA cohort, https://www.ebi.ac.uk/ena/browser/view/PRJEB52891) and PRJEB38481 (RIFSYS cohort, https://www.ebi.ac.uk/ena/browser/view/PRJEB38481), and from https://www.microbiomeatlas.org under the project accession PRJEB38483 (healthy cohort, https://www.ebi.ac.uk/ena/browser/view/PRJEB38483). Metagenomic and metabolomic data for the TIPS cohort from the NEPTUN study (NCT03628807, https://clinicaltrials.gov/study/NCT03628807) can be available upon request via the European Association for the Study of the Liver (EASL). Genome‐scale metabolic models corresponding to the MSPs can be obtained from the Microbiome Atlas website (https://www.microbiomeatlas.org). The full summary statistics to support the findings of this study are included within the supplementary information files. The data used for the figures and scripts used in this study can be found on GitHub: https://github.com/sysbiomelab/Oral-gut-liver. Supplementary materials (methods, figures, tables, graphical abstract, slides, videos, Chinese translated version, and updated materials) may be found in the online DOI or iMeta Science http://www.imeta.science/. The data that support the findings of this study are available in the supplementary material of this article.
