# Supplementary material for: Integrative host–microbiome modeling uncovers the implication of oral–gut translocation in advanced cirrhosis
Source: Imeta. 2026 May 7;5(3):e70131. doi: 10.1002/imt2.70131 (PMC13377410; doi:10.1002/imt2.70131)
Supplement: Supplementary file 1 — Figure S1. Determination of gut and oral reactotypes. Figure S2. Association of gut and oral reactotypes with clinical and demographic characteristics. Figure S3. Unique reactions featured in each gut and oral reactotype. Figure S4. Significant reactions distinguishing low‐ and high‐OGMD patients. Figure S5. Clinical metadata and aetiology differences between high‐ and low‐OGMD patients. Figure S6. Linear regression analysis of tMSPs, remaining overlapping MSPs and other MSPs in relation to OGMD. Figure S7. Flux balance and variability analysis of translocation species and healthy‐gut commensals. Figure S8. Metabolic reactions involved in NH3 production of tMSPs. Figure S9. Subsystem‐level metabolic differences between cirrhotic and healthy liver GSMMs under increasing NH3 concentrations. [file IMT2-5-e70131-s001.docx]

**Supporting information to**

**Integrative host-microbiome modelling uncovers the implication of oral-gut translocation in advanced cirrhosis**

**Running title:** Oral-gut-liver modelling in cirrhosis

Yi Jin^1^, Frederick Clasen^1^, Fernando Garcia-Guevara^1^, Sania Arif^2^, Robert Schierwagen^2^, Gholamreza Bidkhori^1^, Michael Praktiknjo^2^, Maximilian J. Brol^2^, Frank E. Uschner^2^, Florence A. Castellia^3^, Nicolas Pons^4^, Benoit Quinquis^4^, Nathalie Galleron^4^, Kevin Da Silva^4^, Christophe Junot^3^, Debbie L. Shawcross^5^, David L. Moyes^1^, Rajiv Jalan^6,7^, S. Dusko Ehrlich^8^, Vishal C. Patel^5^, Jonel Trebicka^2,7,9^, Saeed Shoaie^1,10*^

^1^ Centre for Host-Microbiome Interactions, Faculty of Dentistry, Oral & Craniofacial Sciences, King’s College London, SE1 9RT, United Kingdom.

^2^ Department of Internal Medicine B, University of Münster, 48149 Münster, Germany.

^3^ Université Paris-Saclay, CEA, INRAE, Département Médicaments et Technologies pour la Santé (MTS), MetaboHUB-IDF, 91191 Gif-sur-Yvette, France.

^4^ Université Paris-Saclay, INRAE, MGP, 78350, Jouy-en-Josas, France.

^5^ Roger Williams Institute of Liver Studies, School of Immunology and Microbial Sciences, Faculty of Life Sciences and Medicine, King’s College London, London, SE5 8AF, United Kingdom.

^6^ Liver Failure Group, UCL Institute for Liver and Digestive Health, Upper third floor, Royal Free Campus, Rowland Hill Street, Hampstead, London, NW3 2PF, United Kingdom.

^7^ European Foundation for the Study of Chronic Liver Failure, EF CLIF, 08036 Barcelona, Spain.

^8^ Department of Clinical and Movement Neurosciences, UCL Queen Square Institute of Neurology, University College London, London, WC1N 3BG, United Kingdom.

^9^ Department of Medical Gastroenterology and Hepatology, University of Southern Denmark, 5230 Odense, Denmark.

^10^ Quantitative Systems Biology, Faculty of Medicine, Biruni University, Istanbul 34015, Turkey.

* Corresponding author: saeed.shoaie@kcl.ac.uk (Saeed Shoaie)

**Supplementary methods**

**Study cohorts and clinical data**

This study utilised two previously published cirrhosis cohorts with paired oral and stool metagenomes: RIFSYS study (Patel *et al.* [1]) and the GLA study (Lee *et al.* [2]). Patients from both cohorts were recruited at King’s College Hospital NHS Foundation Trust, London, under defined inclusion/exclusion criteria, with demographic and clinical data (age, sex, BMI, MELD score) available as described in the original publications. The RIFSYS cohort contributed 35 patients with cirrhosis at baseline (as defined in [1]), and the GLA cohort contributed 50 patients with cirrhosis and 13 healthy controls, all providing paired saliva and stool samples. An additional healthy control cohort from the UK (*n* = 48) [3] was included to expand the control group. Ethical approvals and written informed consent were obtained as reported in the original studies [1,2]. The metagenomic datasets analysed in this study were obtained from previously published cohorts, and detailed descriptions of the study populations, including demographic characteristics and clinical metadata, have been reported in the original studies [1,2].

For further interpretation of the impact on host-microbiome interactions, a cohort of 34 patients with acutely decompensated cirrhosis was used, which was recruited at the Department of Internal Medicine I, University Clinic Bonn, Germany, as part of the NEPTUN study (NCT03628807). All patients underwent transjugular intrahepatic portosystemic shunt (TIPS) insertion due to complications including ascites, hepatic encephalopathy, gastrointestinal bleeding, or bacterial infection. Portal vein serum samples were collected during TIPS, and 113 stool samples from 34 patients were obtained post-procedure. Demographic and clinical information (sex, age, disease aetiology, severity scores) was recorded at baseline. All patients provided written informed consent, and the study was approved by the local ethics committee (University Hospital Bonn, approval no. 203/13).

**Blood metabolomics**

As part of the NEPTUN study, serum metabolomics data were generated. Methanol precipitation [4] was used for the extraction of metabolites and analysed by liquid chromatography coupled to high-resolution mass spectrometry (LC-HRMS) using an Ultimate 3000 chromatographic system and Exactive mass spectrometer (Thermo Fisher Scientific) with electrospray ionisation. Data were acquired using a C18 column, with a MS detection in the positive ionization mode and using a ZIC-pHILIC column with a MS detection in the negative ion mode, both in a single analytical batch. Pooled quality-control samples, prepared by combining aliquots from all study samples, were injected at regular intervals to monitor instrument performance.

Raw data were processed with the Workflow4Metabolomics platform [5] (https://workflow4metabolomics.org/), and features were annotated against an in-house spectral database using accurate mass and retention time [6−8]. Signals were retained if they met standard quality thresholds (correlation with QC dilution factors > 0.7, coefficient of variation < 30% across QC samples, and signal-to-blank ratio > 3). Peaks of annotated metabolites were manually inspected in QC samples using Xcalibur software.

Metabolite identities were confirmed by LC-MS/MS on a Q-Exactive mass spectrometer against authentic standards, following Metabolomics Standards Initiative recommendations requiring at least two orthogonal criteria (accurate mass, isotopic pattern, MS/MS spectrum, or retention time). The resulting dataset contained m/z and retention time values with semi-quantitative abundances expressed as chromatographic peak areas [9].

**Stool metagenomics**

Metagenomic Species Pan-genomes (MSP) abundance tables generated in the original publications were used for downstream analyses [1−3]. These tables were obtained using the METEOR pipeline (https://forgemia.inra.fr/metagenopolis/meteor), a quantitative metagenomic profiling approach that maps trimmed sequencing reads to oral and gut microbial gene catalogues for saliva and faecal samples, respectively, to estimate gene counts per sample [10]. Specifically, gene counts were rarefied to 20 million reads per sample (RIFSYS saliva and faeces, GLA faeces) and to 10 million reads per sample (GLA saliva) to minimize variability in sequencing depth and normalized based on gene length and total counts prior to MSP abundance estimation. Samples with sequencing depth below these thresholds were excluded, leaving 53 saliva samples from cirrhosis patients, 56 saliva samples from healthy controls, 85 faecal samples from cirrhosis patients, and 61 faecal samples from healthy controls for downstream analysis (LC-faeces: *n* = 85, LC-saliva: *n* = 53, HC-faeces: *n* = 61, HC-saliva: *n* = 56).

For the NEPTUN study, DNA was extracted from stool samples following IHMS guidelines (SOP 07 V2 H). Library preparation was performed using the Ion Plus Fragment Library Kit (Thermo Fisher Scientific), and sequencing was conducted on the Ion Proton platform, generating an average of 25.9 ± 9.5 million single-end 150 bp reads per sample. Reads were quality-filtered using Alien Trimmer v0.4.0 [11] and mapped against the human genome (GRCh38-p13) with Bowtie2 v2.3.4 [12] to remove host contamination. High-quality reads were aligned against the Integrated Gene Catalogue (IGC2) reference catalogue [13] using Bowtie2 with a 95% identity threshold implemented in the METEOR v3.2 pipeline [10]. Gene abundance tables were generated by unique read mapping, with shared reads allocated according to relative unique mapping counts. Taxonomic assignment was performed using Kraken2 [14,15], and MSPs were quantified in line with the discovery cohorts.

**Determining relative reaction abundance and reactobiome analysis**

To collect the metabolic features, the normalised gene count table from saliva and faecal samples was mapped against KEGG reaction identifications through orthologs. To determine the relative abundance of each reaction within the oral and gut microbiota, we constructed the reactobiome matrix, representing the cumulative abundance of each reaction [16]. The relative abundance of a reaction 𝑖 in each sample 𝑗 (𝑅_𝑖𝑗_) was calculated by summing the reaction count (𝑟_𝑖k_) contributed by all MSPs in each sample:

$$R_{ij}=\sum_{k=1}^{n} (r_{ik}\times A_{kj})$$

Where:

𝑛: The total number of MSPs in sample 𝑗.

𝑅_𝑖𝑗_: The relative abundance of reaction 𝑖 in sample 𝑗.

𝑟_𝑖𝑘_: The count of reaction 𝑖 in MSP 𝑘 (from the reaction count table).

𝐴_𝑘𝑗_: The abundance of MSP 𝑘 in sample 𝑗.

Reactotypes were identified for the oral and gut reactobiome using unsupervised clustering based on reactobiome profiles. Specifically, clustering was performed using the Dirichlet multinomial mixtures model, implemented in the R package *DirichletMultinomial*, to determine the optimal number of clusters (parameter k = 1:5) for both oral and gut reactotypes [17]. To account for differences in bacterial abundance, we normalised the relative reaction abundance ("reactobiome") based on the presence or absence of each reaction in each sample [16]. The normalised reactobiome was calculated by expressing the presence (𝑝_𝑖𝑘_ = 1) or absence (𝑝_𝑖𝑘_ = 0) of each reaction as a count per 500 MSPs for each sample:

$$R_{ij}^{'}=\left( \frac{\sum_{k=1}^{n} \left( p_{ik}\times A_{kj} \right)}{n} \right)\times500$$

Where:

𝑅′_𝑖𝑗_: The normalised relative abundance of reaction 𝑖 in sample 𝑗.

𝑝_𝑖𝑘_: Presence (1) or absence (0) of reaction 𝑖 in MSP 𝑘.

𝐴_𝑘𝑗_: The abundance of MSP 𝑘 in sample 𝑗.

𝑛: The total number of MSPs considered.

The number of Dirichlet components (reactotypes) of oral and gut were determined by fitting Dirichlet mixtures and Laplace approximation to the model using the function *dmm* and *laplace* with minimising the Laplace score. The gut reactobiome model fitting yielded positive infinite values at k=4 and k=5, suggesting a significant approximation error (Figure S1). Consequently, these values were deemed unsuitable for further consideration in line with the goal to minimise the Laplace score. The most important drivers of each reactotype were identified by ranking variables (reactions) based on assignment strength and filtering for those with absolute values exceeding the 99th percentile, focusing on the top 1% of contributors for each cluster, followed by retaining only those drivers specific to a single reactotype to ensure the identification of reactotype-specific features. We investigated the differences in metadata among the reactotypes using the Kruskal-Wallis test for three gut reactotypes, and the Wilcoxon rank-sum test for pairwise comparison between reactotypes for oral and gut separately. Statistical analysis was performed using R software version 4.3.0.

**Single-species microbial metabolic modelling**

Gut microbiome microbial genome-scale metabolic models (GSMMs) were downloaded from Microbiome Atlas (https://www.microbiomeatlas.org/) with matched MSP identifiers [3]. To explore the metabolic capacities of individual microbes, we applied single-species modelling. Nutrient availability for single-species metabolic modelling was defined using the *DietConstrain* function of the MIGRENE toolbox [16], with the *dietNumber* parameter specifying five distinct dietary regimes: high-fibre plant-based, high-fibre omnivore, high-protein plant-based, high-protein omnivore, and the UK average diet. Steady-state metabolic fluxes for all individual microbial models were inferred using Flux Balance Analysis (FBA), which optimises a maximising objective function (biomass production) under defined stoichiometric constraints. To further investigate the flexibility of flux distributions, Flux Variability Analysis (FVA) was performed, identifying the maximum feasible flux production for each metabolite. These simulations were processed using the COnstraint-Based Reconstruction and Analysis (COBRA) toolbox v.3.0 implemented in MATLAB R2023a [18,19].

**Community microbial metabolic model reconstruction and simulations**

Two representative gut community metabolic models were reconstructed to compare the community-level metabolic difference of high- and low-OGMD patients. Community models were constructed with the top 15 most abundant MSPs for each group to maintain computational feasibility. The most abundant and prevalent MSPs of each group were identified based on a minimum prevalence threshold of 0.5, and the MSPs meeting this threshold were then ranked according to their average relative abundance within each group to get the top 15 abundant MSPs. Community models were generated using the *MakeCommunity* function from the MIGRENE toolbox using the MSP average relative abundance profiles, with the objective function set to maximise the biomass of the entire community [16]. This function simulates collective growth by aggregating the biomass reactions of individual species, with stoichiometric coefficients weighted by their relative abundance, ensuring that more abundant species contribute more significantly to overall biomass. FBA was employed for community model optimisation. Nutrient availability was constrained based on the UK average diet, and anaerobic conditions were imposed by constraining oxygen availability to zero.

To further simulate the community-level metabolic capabilities of the 16 co-enriched MSPs in the gut, we first reconstructed community models for both high- and low-OGMD patient groups using the top 15 most abundant MSPs in each group, however, excluding the 16 co-enriched MSPs. These models served as the base models. Subsequently, the 16 co-enriched MSPs were added individually to each community model, resulting in a total of 17 community models for each OGMD group. FBA was employed to predict metabolic fluxes for all community models under the same simulation conditions.

**Human tissue metabolic model reconstruction and simulation**

We reconstructed context-specific human liver genome-scale metabolic models (GSMMs) for healthy and cirrhotic liver using bulk transcriptomes from the GepLiver Bulk 20 cohort [20]. Gene identifiers were harmonised to the name used by the template human liver GSMM generated from Human-GEM (Zenodo record 3583004) and mapped to the model’s gene-protein-reaction (GPR) rules [21]. We computed the mean expression per gene within each condition (healthy or cirrhosis) and used those condition-level means for model constraining.

We applied an adapted E-flux procedure to derive condition-specific flux bounds from gene expression [22,23]. For each reaction, we computed a condition-specific expression score from the model’s GPR. For reactions associated with a single gene, the mean expression level of that gene across biological replicates within each condition was used to determine the corresponding flux bound. In cases where multiple genes were linked by an “OR” relationship (representing isoenzymes), the bound was calculated as the sum of the mean expression levels of all associated genes. For reactions linked by an “AND” relationship (representing protein complexes), the bound was set to the lowest mean expression value among the constituent genes, reflecting the limiting subunit in the complex. Additionally, to investigate ammonia metabolism, we constrained the flux of extracellular NH_3_ uptake sequentially to 0, 0.1, 0.5, 1, 2, and 5 mmol gDW^-1^ h^-1^, while blocking NH_4_^+^ production.

Brain and muscle were represented by generic Human-GEM tissue GSMMs, in which we varied the upper bounds on extracellular NH_3_ and acetate uptake from 0 to 100 mmol gDW^-1^ h^-1^ (0, 0.1, 0.5, 1, 2, 5, 10, 20, 30, 40, 50, 100). All simulations were performed with the RAVEN 2.0 toolbox [24] using *solveLP* for FBA, optimising the model’s biomass reaction as the objective.

**Statistical analysis**

Statistical analyses were performed in R (version 4.3.0) unless otherwise stated. Differentially abundant microbial species and metabolic reactions were identified using Wilcoxon rank-sum tests, with *p*-values adjusted for multiple comparisons using the Benjamini-Hochberg false discovery rate (FDR). The statistical result of species abundance is shown in Table S7.

Translocation-associated metagenomic species pangenomes (tMSPs) were identified based on their relative abundance patterns and presence across oral and gut microbiomes. Briefly, species with higher mean abundance in low-OGMD patients compared to high-OGMD patients were identified separately in the gut (*n* of species = 459) and oral (*n* of species = 170) microbiomes. The intersection of these two sets yielded 16 overlapping species. The intersection of these sets yielded 16 overlapping species, corresponding to species present in both sites and exhibiting higher mean abundance in low-OGMD patients across oral and gut microbiomes. The remaining MSPs shared between oral and gut microbiomes (*n* of species = 19), which did not meet this criterion, were defined as other oral-gut overlapping MSPs. Healthy gut commensals were defined as species with a prevalence greater than 10% across healthy samples and that were significantly enriched in health compared with cirrhosis (Wilcoxon rank-sum test, Benjamini-Hochberg correction, FDR < 0.001, Log_2_FC > 3.5).

Prevalence of translocation-associated metagenomic species pangenomes (tMSPs) was calculated separately for oral and gut microbiomes using species-level relative abundance tables, defined as the proportion of samples in which the species was detected (relative abundance > 0). Sample-level relative abundance of the MSPs was analysed in relation to OGMD using linear regression (*lm* in R), with OGMD as a continuous independent variable. To quantify the relative association of each tMSP with oral versus gut microbiomes, an oral-origin score was defined as $\text{Prevalence}_{\text{oral}}\text{/(}\text{Prevalence}_{\text{oral}}\text{+}\text{Prevalence}_{\text{gut}}\text{)}$.

Correlations between microbial features and clinical parameters, including MELD scores, were evaluated by Spearman correlation analysis. The association between OGMD with MELD scores were evaluated by linear regression using the *lm* function. Multigroup comparisons, such as differences among reactotypes, were tested by Kruskal-Wallis followed by pairwise Wilcoxon rank-sum tests where appropriate. Spearman correlation coefficients were calculated between microbial relative abundances and normalised serum metabolite levels. Correlation *p*-values were adjusted using the Benjamini–Hochberg false discovery rate (FDR) correction.

Bray-Curtis dissimilarities were calculated using the *vegan* package to quantify oral-gut metabolic distances (OGMD), and differences between high- and low-OGMD groups were assessed using Wilcoxon rank-sum tests. Differences in liver disease aetiology between high- and low-OGMD groups were assessed using a two-sided Fisher’s exact test in R. Fisher’s exact test was performed to evaluate whether the distribution of aetiology categories differed between groups, and the two-sided *p*-value was reported. For metabolic modelling, predicted flux values were compared between groups using Wilcoxon rank-sum tests. Unless otherwise stated, all reported *p*-values are two-sided, and results with FDR < 0.05 were considered statistically significance, while FDR ≤ 0.1 was considered suggestive of a potential association.

**Extended discussion, study limitations and perspectives**

Several host and environmental factors may influence oral-gut microbial interactions in cirrhosis and should be considered when interpreting these findings. Not in the least because of the predictive modelling nature of our study but also because of limitations of the cohorts used.

Alcohol consumption represents a major aetiological factor in cirrhosis and may influence both host metabolism and gut microbial composition [25]. Ethanol metabolism is an important source of host-derived acetate and may influence interpretations of microbial acetate production [26]. Comparative analyses across cirrhosis aetiologies may therefore help to determine whether alcohol-related liver disease exhibits distinct patterns of microbial translocation or enhanced acetate-ammonia metabolic activity relative to other cirrhosis types. Other microbial metabolic pathways, including metabolism of other short-chain fatty acids and endotoxin-associated inflammatory responses, may also contribute to systemic metabolic alterations in cirrhosis and merit further investigation [27,28].

Oral health status was not systematically recorded across the analysed cohorts and therefore could not be incorporated into the present analyses. Oral diseases, particularly periodontitis, are known to strongly influence oral microbial community composition and have been linked to microbial dissemination beyond the oral cavity and systemic inflammation [29−31]. Diet and medication use may also influence microbiome composition but were not fully controlled for in the cohorts re-analysed in our study and may represent additional sources of variation [32]. Notably, commonly prescribed cirrhosis medications such as proton pump inhibitors can alter microbial communities and may promote microbial dissemination [33]. Integrating oral clinical assessments together with paired oral-gut microbiome profiling will help clarify the role of oral health and lifestyle factors in microbial transmission along the gastrointestinal tract.

More broadly, the metabolic outputs reported here were inferred from genome-scale metabolic modelling and therefore represent predicted metabolic capabilities. The primary analyses were performed in a single-centre cohort with paired oral and gut metagenomes, while an independent dataset confirmed the increased prevalence of oral-related translocation-associated species in gut microbiomes but lacked paired oral-gut samples, preventing further evaluation of OGMD dynamics across datasets. Larger multi-cohort datasets, particularly those including longitudinal sampling, will enable further assessment of OGMD and may help determine its potential utility as a microbiome-based biomarker, including the definition of clinically relevant thresholds [34].

The ecological mechanisms enabling oral-derived microbes to establish within the gut microbiome remain incompletely understood. Altered gut environmental conditions in cirrhosis, including changes in nutrient availability and colonization resistance, may create ecological niches favourable for oral taxa such as *Prevotella* and *Veillonella*. Future studies integrating longitudinal sampling, targeted metabolomics and experimental microbiome models will help clarify the mechanisms and functional consequences of oral-gut microbial transmission in cirrhosis.

To further interpret the increased susceptibility of low-OGMD communities to oral-derived taxa, several ecological features may be considered. First, enhanced amino-acid fermentation observed in these communities may increase the local availability of nitrogenous substrates, thereby providing a metabolically favourable niche for translocating taxa with ammonia-producing potential. Second, reduced colonization resistance may arise from the depletion of commensal gut microbes, particularly short-chain fatty acid (SCFA)-producing taxa such as butyrate producers, which are known to contribute to epithelial barrier maintenance and microbial inhibition [35,36]. Loss of these functions may facilitate the persistence and metabolic activity of oral-derived species in the gut environment [30,33]. Third, alterations in microenvironmental conditions, including pH and redox potential, may further support the adaptation of oral taxa following translocation [37].

In addition to these ecological factors, gut barrier dysfunction in cirrhosis may promote the translocation of microbial-associated molecules such as lipopolysaccharide (LPS), contributing to endotoxemia and systemic inflammation [38,39]. These inflammatory processes may act in concert with microbial metabolic outputs, linking community restructuring to host metabolic disturbance. Within this broader context, the enrichment of ammonia-producing pathways, together with the co-production of acetate observed in several translocating taxa, may represent one functional consequence of oral-gut microbial convergence rather than an isolated mechanism. Overall, these considerations support a model in which ecological disruption, metabolic adaptation, and host-microbiome interactions jointly shape the impact of oral-gut translocation in cirrhosis.

**REFERENCES**

1. Patel, Vishal C., Sunjae Lee, Mark J.W. McPhail, Kevin Da Silva, Susie Guilly, Ane Zamalloa, Elizabeth Witherden, et al. 2022. “Rifaximin-α reduces gut-derived inflammation and mucin degradation in cirrhosis and encephalopathy: RIFSYS randomised controlled trial.” *Journal of Hepatology* 76: 332–342. <https://doi.org/10.1016/j.jhep.2021.09.010>

2. Lee, Sunjae, Bethlehem Arefaine, Neelu Begum, Marilena Stamouli, Elizabeth Witherden, Merianne Mohamad, Azadeh Harzandi, et al. 2024. “Oral-gut microbiome interactions in advanced cirrhosis: characterisation of pathogenic enterotypes and salivatypes, virulence factors and antimicrobial resistance.” *Journal of Hepatology* 82: 622–633. <https://doi.org/10.1016/j.jhep.2024.09.046>

3. Lee, Sunjae, Theo Portlock, Emmanuelle Le Chatelier, Fernando Garcia-Guevara, Frederick Clasen, Florian Plaza Oñate, Nicolas Pons, et al. 2024. “Global compositional and functional states of the human gut microbiome in health and disease.” *Genome Research* 34: 967–978. <https://doi.org/10.1101/gr.278637.123>

4. Moreau, Richard, Joan Clària, Ferran Aguilar, François Fenaille, Juan José Lozano, Christophe Junot, Benoit Colsch, et al. 2020. “Blood metabolomics uncovers inflammation-associated mitochondrial dysfunction as a potential mechanism underlying ACLF.” *Journal of Hepatology* 72: 688–701. <https://doi.org/10.1016/j.jhep.2019.11.009>

5. Giacomoni, Franck, Gildas Le Corguillé, Misharl Monsoor, Marion Landi, Pierre Pericard, Mélanie Pétéra, Christophe Duperier, et al. 2015. “Workflow4Metabolomics: a collaborative research infrastructure for computational metabolomics.” *Bioinformatics* 31: 1493–1495. <https://doi.org/10.1093/bioinformatics/btu813>

6. Weiss, Nicolas, Pierre Barbier Saint Hilaire, Benoit Colsch, Foucauld Isnard, Suleiman Attala, Augustin Schaefer, Maria del Mar Amador, et al. 2016. “Cerebrospinal fluid metabolomics highlights dysregulation of energy metabolism in overt hepatic encephalopathy.” *Journal of Hepatology* 65: 1120–1130. <https://doi.org/10.1016/j.jhep.2016.07.046>

7. Boudah, Samia, Marie-Françoise Olivier, Sandrine Aros-Calt, Lydie Oliveira, François Fenaille, Jean-Claude Tabet, Christophe Junot. 2014. “Annotation of the human serum metabolome by coupling three liquid chromatography methods to high-resolution mass spectrometry.” *Journal of Chromatography B* 966: 34–47. <https://doi.org/https://doi.org/10.1016/j.jchromb.2014.04.025>

8. Roux, Aurelie, Ying Xu, Jean-François Heilier, Marie-Françoise Olivier, Eric Ezan, Jean-Claude Tabet, Christophe Junot. 2012. “Annotation of the human adult urinary metabolome and metabolite identification using ultra high performance liquid chromatography coupled to a linear quadrupole ion trap-Orbitrap mass spectrometer.” *Analytical Chemistry* 84: 6429–6437. <https://doi.org/10.1021/ac300829f>

9. Sumner, Lloyd W., Alexander Amberg, Dave Barrett, Michael H. Beale, Richard Beger, Clare A. Daykin, Teresa W. M. Fan, et al. 2007. “Proposed minimum reporting standards for chemical analysis Chemical Analysis Working Group (CAWG) Metabolomics Standards Initiative (MSI).” *Metabolomics* 3: 211–221. <https://doi.org/10.1007/s11306-007-0082-2>

10. Ghozlane, Amine, Florence Thirion, Florian Plaza Oñate, Franck Gauthier, Emmanuelle Le Chatelier, Anita Annamalé, Mathieu Almeida, Stanislav D. Ehrlich, Nicolas Pons. 2025. “Accurate profiling of microbial communities for shotgun metagenomic sequencing with Meteor2.” *Microbiome* 13: 227. <https://doi.org/10.1186/s40168-025-02249-w>

11. Criscuolo, Alexis, Sylvain Brisse. 2014. “AlienTrimmer removes adapter oligonucleotides with high sensitivity in short-insert paired-end reads. Commentary on Turner (2014) Assessment of insert sizes and adapter content in FASTQ data from NexteraXT libraries.” *Frontiers in Genetics* 5: 130. <https://doi.org/10.3389/fgene.2014.00130>

12. Langmead, Ben, Steven L. Salzberg. 2012. “Fast gapped-read alignment with Bowtie 2.” *Nature Methods* 9: 357–359. <https://doi.org/10.1038/nmeth.1923>

13. Li, Junhua, Huijue Jia, Xianghang Cai, Huanzi Zhong, Qiang Feng, Shinichi Sunagawa, Manimozhiyan Arumugam, et al. 2014. “An integrated catalog of reference genes in the human gut microbiome.” *Nature Biotechnology* 32: 834–841. <https://doi.org/10.1038/nbt.2942>

14. Wood, Derrick E., Jennifer Lu, Ben Langmead. 2019. “Improved metagenomic analysis with Kraken 2.” *Genome Biology* 20: 257. <https://doi.org/10.1186/s13059-019-1891-0>

15. Wood, Derrick E., Steven L. Salzberg. 2014. “Kraken: ultrafast metagenomic sequence classification using exact alignments.” *Genome Biology* 15: R46. <https://doi.org/10.1186/gb-2014-15-3-r46>

16. Bidkhori, Gholamreza, Saeed Shoaie. 2024. “MIGRENE: The Toolbox for Microbial and Individualized GEMs, Reactobiome and Community Network Modelling.” *Metabolites* 14: 132. <https://doi.org/10.3390/metabo14030132>

17. Holmes, Ian, Keith Harris, Christopher Quince. 2012. “Dirichlet Multinomial Mixtures: Generative Models for Microbial Metagenomics.” *PLOS ONE* 7: e30126. <https://doi.org/10.1371/journal.pone.0030126>

18. Heirendt, Laurent, Sylvain Arreckx, Thomas Pfau, Sebastián N. Mendoza, Anne Richelle, Almut Heinken, Hulda S. Haraldsdóttir, et al. 2019. “Creation and analysis of biochemical constraint-based models using the COBRA Toolbox v.3.0.” *Nature Protocols* 14: 639–702. <https://doi.org/10.1038/s41596-018-0098-2>

19. The MathWorks, Inc. 2023. 'MATLAB R2023a', The MathWorks, Inc., Natick, Massachusetts.

20. Li, Ziteng, Hena Zhang, Qin Li, Wanjing Feng, Xiya Jia, Runye Zhou, Yi Huang, et al. 2023. “GepLiver: an integrative liver expression atlas spanning developmental stages and liver disease phases.” *Scientific Data* 10: 376. <https://doi.org/10.1038/s41597-023-02257-1>

21. Robinson, Jonathan L., Pınar Kocabaş, Hao Wang, Pierre-Etienne Cholley, Daniel Cook, Avlant Nilsson, Mihail Anton, et al. 2020. “An atlas of human metabolism.” *Science Signaling* 13: eaaz1482. https://doi.org/10.1126/scisignal.aaz1482

22. Colijn, Caroline, Aaron Brandes, Jeremy Zucker, Desmond S. Lun, Brian Weiner, Maha R. Farhat, Tan-Yun Cheng, D. Branch Moody, Megan Murray, James E. Galagan. 2009. “Interpreting Expression Data with Metabolic Flux Models: Predicting Mycobacterium tuberculosis Mycolic Acid Production.” *PLOS Computational Biology* 5: e1000489. <https://doi.org/10.1371/journal.pcbi.1000489>

23. Clasen, Frederick, Patrícia M. Nunes, Gholamreza Bidkhori, Nourdine Bah, Stefan Boeing, Saeed Shoaie, Dimitrios Anastasiou. 2023. “Systematic diet composition swap in a mouse genome-scale metabolic model reveals determinants of obesogenic diet metabolism in liver cancer.” *iScience* 26: <https://doi.org/10.1016/j.isci.2023.106040>

24. Wang, Hao, Simonas Marcišauskas, Benjamín J. Sánchez, Iván Domenzain, Daniel Hermansson, Rasmus Agren, Jens Nielsen, Eduard J. Kerkhoven. 2018. “RAVEN 2.0: A versatile toolbox for metabolic network reconstruction and a case study on Streptomyces coelicolor.” *PLOS Computational Biology* 14: e1006541. <https://doi.org/10.1371/journal.pcbi.1006541>

25. Roerecke, Michael, Afshin Vafaei, Omer S.M. Hasan, Bethany R. Chrystoja, Marcus Cruz, Roy Lee, Manuela G. Neuman, Jürgen Rehm. 2019. “Alcohol Consumption and Risk of Liver Cirrhosis: A Systematic Review and Meta-Analysis.” *The American Journal of Gastroenterology* 114: 1574–1586. <https://doi.org/10.14309/ajg.0000000000000340>

26. Martino, Cameron, Livia S. Zaramela, Bei Gao, Mallory Embree, Janna Tarasova, Seth J. Parker, Yanhan Wang, et al. 2022. “Acetate reprograms gut microbiota during alcohol consumption.” *Nature Communications* 13: 4630. <https://doi.org/10.1038/s41467-022-31973-2>

27. Bajaj, J. S. 2019. “Alcohol, liver disease and the gut microbiota.” *Nat Rev Gastroenterol Hepatol* 16: 235–246. <https://doi.org/10.1038/s41575-018-0099-1>

28. Betrapally, Naga S., Patrick M. Gillevet, Jasmohan S. Bajaj. 2017. “Gut microbiome and liver disease.” *Translational Research* 179: 49–59. <https://doi.org/10.1016/j.trsl.2016.07.005>

29. Albuquerque-Souza, Emmanuel, Sinem E. Sahingur. 2022. “Periodontitis, chronic liver diseases, and the emerging oral-gut-liver axis.” *Periodontology 2000* 89: 125–141. <https://doi.org/10.1111/prd.12427>

30. Acharya, Chathur, Sinem Esra Sahingur, Jasmohan S. Bajaj. 2017. “Microbiota, cirrhosis, and the emerging oral-gut-liver axis.” *JCI Insight* 2: e94416. <https://doi.org/10.1172/jci.insight.94416>

31. Santamaria, Pasquale, Yi Jin, Mandeep Ghuman, Saeed Shoaie, David Spratt, Giuseppe Troiano, Luigi Nibali. 2024. “Microbiological and molecular profile of furcation defects in a population with untreated periodontitis.” *Journal of Clinical Periodontology* 51: 1421–1432. <https://doi.org/10.1111/jcpe.14034>

32. David, Lawrence A., Corinne F. Maurice, Rachel N. Carmody, David B. Gootenberg, Julie E. Button, Benjamin E. Wolfe, Alisha V. Ling, et al. 2014. “Diet rapidly and reproducibly alters the human gut microbiome.” *Nature* 505: 559–563. <https://doi.org/10.1038/nature12820>

33. Bajaj, Jasmohan S., Douglas M. Heuman, Phillip B. Hylemon, Arun J. Sanyal, Melanie B. White, Pamela Monteith, Nicole A. Noble, et al. 2014. “Altered profile of human gut microbiome is associated with cirrhosis and its complications.” *Journal of Hepatology* 60: 940–947. <https://doi.org/10.1016/j.jhep.2013.12.019>

34. Ballester, Maria Pilar, Thomas H. Tranah, Lorenz Balcar, Alessandra Fiorillo, Javier Ampuero, Annarein J. C. Kerbert, Karen L. Thomsen, et al. 2023. “Development and validation of the AMMON-OHE model to predict risk of overt hepatic encephalopathy occurrence in outpatients with cirrhosis.” *Journal of Hepatology* 79: 967–976. <https://doi.org/10.1016/j.jhep.2023.05.022>

35. Pohl, Keith, Prebashan Moodley, Ashwin Dhanda. 2022. “The effect of increasing intestinal short-chain fatty acid concentration on gut permeability and liver injury in the context of liver disease: A systematic review.” *Journal of Gastroenterology and Hepatology* 37: 1498–1506. <https://doi.org/10.1111/jgh.15899>

36. Bloom, Patricia P., Jesús M. Luévano, Kelsey J. Miller, Raymond T. Chung. 2021. “Deep stool microbiome analysis in cirrhosis reveals an association between short-chain fatty acids and hepatic encephalopathy.” *Annals of Hepatology* 25: 100333. <https://doi.org/10.1016/j.aohep.2021.100333>

37. Kunath, Benoit J., Charlotte De Rudder, Cedric C. Laczny, Elisabeth Letellier, Paul Wilmes, Benoit J. Kunath, Charlotte De Rudder, Cedric C. Laczny, Elisabeth Letellier, Paul Wilmes. 2024. “The oral–gut microbiome axis in health and disease.” *Nature Reviews Microbiology* 22: 791–805. <https://doi.org/10.1038/s41579-024-01075-5>

38. Tsiaoussis, Georgios I, Stelios F Assimakopoulos, Athanassios C Tsamandas, Christos K Triantos, Konstantinos C Thomopoulos. 2015. “Intestinal barrier dysfunction in cirrhosis: Current concepts in pathophysiology and clinical implications.” *World Journal of Hepatology* 7: 2058–2068. <https://doi.org/10.4254/wjh.v7.i17.2058>

39. Di Vincenzo, Federica, Angelo Del Gaudio, Valentina Petito, Loris Riccardo Lopetuso, Franco Scaldaferri. 2024. “Gut microbiota, intestinal permeability, and systemic inflammation: a narrative review.” *Internal and Emergency Medicine* 19: 275–293. <https://doi.org/10.1007/s11739-023-03374-w>

**Supplementary Figures**

**Figure S1 Determination of gut and oral reactotypes**. (A) Gut reactobiome model fitting. The y-axis represents the Laplace approximation score, with the lowest value indicating k = 3 optimal clusters. (B) Oral reactobiome model fitting. The Laplace score reached its minimum at k = 2.

**Figure S2** **Association of gut and oral reactotypes with clinical and demographic characteristics** (A) Distribution of patient groups across gut reactotypes. (B) Distribution of patient groups across oral reactotypes. (C) BMI across gut reactotypes (G-reto1, G-reto 2, G-reto 3) showed no significant difference (Kruskal-Wallis, *p* = 0.74). (D) Age across gut reactotypes showed a significant overall difference (Kruskal-Wallis, *p* = 0.0051), driven by the G-reto 2 vs G-reto 3 contrast (pairwise Wilcoxon, *p* = 0.0014). (E) BMI across oral reactotypes (O-reto 1, O-reto 2) was not significant (Wilcoxon rank-sum test, *p* = 0.28). (F) Age across oral reactotypes was not significant (Wilcoxon rank-sum test, *p* = 0.53). Abbreviation: G-reto, Gut-reactotype; O-reto, Oral-reactotype.

**Figure S3** **Unique reactions featured in each gut and oral reactotype.** (A) Venn diagram showing the overlap and unique reactions across three gut reactotypes (G-reto 1, G-reto 2, G-reto 3). (B-D) Reactotype-specific reactions enriched in G-reto 1 (B), G-reto 2 (C), and G-reto 3 (D). (E) Venn diagram of oral reactotypes (O-reto 1, O-reto 2). (F and G) Reactotype-specific reactions enriched in O-reto 1 (F) and O-reto 2 (G). Each bar plot shows the relative abundance of the top unique reactions identified as specific drivers of the corresponding reactotype. Abbreviation: G-reto, Gut-reactotype; O-reto, Oral-reactotype.

**Figure S4** **Significant reactions distinguishing low and high-OGMD patients.** (A) Gut reactions identified by Wilcoxon rank-sum test (*p <* 0.05, |log_2_FC| > 2). (B) Oral reactions identified by Wilcoxon rank-sum test (*p <* 0.005, log2FC > 2 or < -1). Bars represent the mean relative abundance of reactions (log scale), with colours indicating low- and high-OGMD groups. Abbreviation: OGMD, Oral-Gut Metabolic Distance.

**Figure S5** **Clinical metadata and aetiology differences between high- and low-OGMD patients.** (A) Comparison of clinical and laboratory metadata between high-OGMD and low-OGMD groups. Boxplots show the median (centre line), interquartile range (box), and 1.5×IQR whiskers; points indicate individual subjects. Two-sided Wilcoxon rank-sum tests were used to assess differences between groups for each variable; nominal *p*-values are shown above each panel. (B) Distribution of liver disease aetiology in high- and low-OGMD groups shown as stacked bar plots (proportion of patients). Overall differences in aetiology composition were assessed using a two-sided Fisher’s exact test (*p* = 0.425). Abbreviation: OGMD, Oral-Gut Metabolic Distance; PSC, Primary Sclerosing Cholangitis; HCV, Hepatitis C virus; SVR, sustained virological response; ALD, Alcohol-associated liver disease; NAFLD, Non-alcoholic fatty liver disease cirrhosis. No major differences were observed between groups for most clinical variables and disease aetiology, suggesting that OGMD stratification is unlikely to be primarily driven by differences in host metadata. Abbreviation: OGMD, Oral-Gut Metabolic Distance.

**Figure S6. Linear regression analysis of tMSPs, remaining overlapping MSPs and other MSPs in relation to OGMD.** (A–B) Linear regression analysis of the summed abundance of the 16 tMSPs against OGMD in gut (A) and oral (B) microbiomes (*p* < 0.05), indicating higher abundance of these species in patients with stronger oral-gut functional convergence. (C–D) Linear regression analysis of the summed abundance of the remaining oral-gut overlapping MSPs (*n* = 19) against OGMD in gut (C) and oral (D) microbiomes (*p* < 0.05). (E–F) Linear regression analysis of the summed abundance of all other MSPs against OGMD in gut (E) and oral (F) microbiomes (*p* < 0.05). Abbreviation: tMSPs, translocation-associated Metagenomic Species Pangenomes; OGMD, Oral-Gut Metabolic Distance.

**Figure S7 Flux balance and variability analysis of translocation species and healthy-gut commensals.** (A) Predicted ammonia (NH_3_) production fluxes of oral-gut translocation-associated species (tMSPs) compared with healthy-gut commensals under a standard UK diet, based on flux balance analysis (FBA) (Wilcoxon rank-sum test, *p* = 0.0086). (B) Predicted nitric oxide (NO) production fluxes of tMSPs versus healthy-gut commensals (Wilcoxon rank-sum test, *p* = 0.00042). (C) Flux variability analysis (FVA) of NH_3_ production showing feasible flux ranges across tMSPs, with most tMSPs displaying NH_3_-producing capacity while the majority of commensals did not. (D and E) Comparative NH_3_ production fluxes f tMSPs and commensals across diets. Y-axes represent predicted fluxes (mmol gDW^-1^ h^-1^). Statistical comparisons were performed using Wilcoxon rank-sum tests; non-significant results are indicated as *p* > 0.05, while significant differences are marked as *p* < 0.05 (*), *p* < 0.01 (**). (F) Predicted nitric oxide (NO) production of single-species models across diets. Genome-scale metabolic models were estimated by flux balance analysis (FBA) under UK average diet (UKD), high-fibre diet (HFD; O, omnivorous; P, plant-based), high-protein diet (HPD; O, omnivorous; P, plant-based), and flux variance analysis (FVA)with UK average diet. Heatmap shows predicted NO flux; bars indicate the number of diets with NO production. (G) Prevalence of translocation-associated MSPs (tMSPs) in oral and gut microbiomes. Scatter plot showing the prevalence of 16 tMSPs in oral and gut microbiomes. Each point represents one tMSP and is labelled with the species name. Prevalence was calculated as the proportion of samples in which the species was detected (relative abundance > 0). Points are coloured by the oral-origin score $\text{Prevalence}_{\text{oral}}\text{/(}\text{Prevalence}_{\text{oral}}\text{+}\text{Prevalence}_{\text{gut}}\text{)}$. The dashed line indicates equal prevalence between oral and gut microbiomes.

**Figure S8 Metabolic reactions involved in NH_3_ production of tMSPs.** (A) Amino acid metabolism pathways contributing to NH_3_ production in single-species genome-scale models. Arrows represent metabolic reactions, with edge labels showing the average weighted flux across species models. Bacterial icons indicate species among 16 translocation species (tMSPs) where the corresponding pathway was observed. (B) Presence and predicted activity of NH_3_-producing pathways across species GSMMs. The heatmap shows flux levels of each reaction, with colours representing predicted flux categories: Very Low (0 **–** 0.001 mmol gDW^-1^ h^-1^), Low (0.001 **–** 0.01), Medium (0.01 **–** 0.1), High (0.1 **–** 0.5), and Very High (> 0.5). The terminal metabolite of each reaction is highlighted in bold. Abbreviation: tMSPs, translocation-associated Metagenomic Species Pangenomes.

**Figure S9 Subsystem-level metabolic differences between cirrhotic and healthy liver GSMMs under increasing NH_3_ concentrations.** (A) Heatmap of metabolic subsystem activity in cirrhosis relative to healthy liver models (log2 fold change, cirrhosis/healthy, |log_2_FC| > 1.5). The columns represent NH3 concentrations constrained, and the y-axis shows metabolic subsystems significantly altered under elevated NH3. Colours indicate log2 fold change in flux values, and rows are annotated by metabolic categories. (B) Heatmap showing subsystems with modest changes (|log_2_FC| ≤ 1.5) in summed absolute fluxes between cirrhotic and healthy liver models. Columns represent simulations under increasing extracellular NH_3_ concentrations (0, 0.1, 0.5, 1, 2, and 5 mmol gDW^-1^ h^-1^). Rows represent metabolic subsystems, coloured by metabolic categories. (C and D) Heatmaps of log_2_ fold changes in summed absolute fluxes for brain (C) and muscle (D) subsystems under increasing extracellular NH_3_ and acetate uptake (0, 0.1, 0.5, 1, 2, 5, 10, 20, 30, 40, 50 and 100 mmol gDW^-1^ h^-1^). Columns show the constrained levels and rows denote metabolic subsystems, with colours indicating relative increases or decreases in flux for each subsystem across the gradient. Abbreviation: GSMM, Genome-Scale Metabolic Model.
